# Tuberculosis in Vertebrates

**Published:** 1889-10

**Authors:** Walter K. Sibely

**Affiliations:** Camb.; From the Pathological Institute of the University of Strassburg, (Prof. Von Recklinghausen)


### Art. XIX.—TUBERCULOSIS IN VERTEBRATES.

By Walter K. Sibley, M.B. Camb.[1]
*From the Pathological Institute of the University of Strassburg*,
(Prof. Von Recklinghausen.)

1. *Tuberculosis in Snakes.*—I have been unable to find in literature any description of tuberculosis in reptiles, with which I could compare the following data ; it therefore needs strong grounds to enable one to describe the interesting case in question as one of true tuberculosis.

An example of (*Tropidonotus matrix* var. *murorum*, a native of Italy) died in a zoological garden after a few months captivity.

Externally, there were on the right side of the body three prominent tumors about the size of hazel-nuts ; these being firmly attached to the ribs, one of them enclosing some seven ribs in its substance. The skin, which was adherent to the tumors, was apparently unchanged. Also numbers of other smaller subcutaneous deposits the size of peas adherent to the skin, but not visible from the exterior, were seen upon removing the cuticle. These occurred generally scattered over the whole extent of the reptile. In addition to these many small nodules were found in the body-cavity along the whole length of the animal on the sides of the vertebral column between the layers of loose fascia here present. These deposits existing in front of the vertebral column along the posterior surface of the lung, are not adherent to the walls of its sac. One at the commencement of the dilatation of the bronchus is, however, firmly adherent to it. Deposits of a similar nature occurred in the corpus adiposus about the kidneys.

There was also a complete chain of small nodules firmly attached to, and often more or less surrounding the upper part of, the aorta, extending from the junction of the two arches on the left side of the body to the lower end of the liver. Along the remainder of its course no other nodules were seen.

[1] This paper originally appeared in German in *Virchow's Archiv*, Bd. 116, 1889, and has now been translated with the kind permission of the Editor. Reprinted from the *Journal of Anatomy and Physiology*. Vol. XXIII.

Upon section, all the deposits appeared to be composed of caseous material.

The *Heart* and *Lungs* presented no obvious lesion.

The *Liver* was slightly enlarged, the capsule not much thickened, and the substance of the organ studded throughout its extent with small nodules mostly the size of a pin's head; others attained the size of peas, and these appeared as caseous when cut. Many of these deposits were readily separated from the liver substance as roundish masses, often with smaller nodules attached to them, and appeared when removed from the liver tissue to be filling and lying in smooth-walled cavities. These deposits were more abundant in the dorsal than the ventral part of the organ. Sometimes two or more small ones were firmly attached to the walls of vessels; these occurred on the branches of the portal vein lying in the liver.

The *Spleen* was found profoundly changed, its tissues being hardly recognisable.

The *Pancreas*, with the naked eye, presented no obvious lesion.

The *Digestive Tract*, especially opposite the lower end of the liver, presented some nodular enlargement in its walls. Some deposit also occurred in the walls of the lower end of the stomach, with thickening of its outer coats.

The *Intestines* were found all firmly matted together by adhesions, the blood-vessels here dilated and full of blood.

In the *Rectum* small nodules occurred in the serous coat, but not implicating the muscular.

No deposits were seen in the *Mesentery* or *Peritoneum*.

The *Kidneys* and the *Reproductive Organs* contained nodules of the same nature as those described above.

MICROSCOPICAL APPERANCES.—The *Liver*, as above mentioned, is studded throughout with deposits, all of which, in section, appear more or less round.

The smallest deposits consist of a collection of small cells with little or no fibrous tissue around them, so that they are not distinctly marked off from the liver cells in their immediate neighborhood. Most of them show foci of degeneration not always occurring in the centre, and sometimes multiple. The larger nodules consist usually of a small central hyaline degenerated area crowded with bacilli, and whose margin are often irregular with processes extending out into the next zone. The central region of this patch appears homogenous. Towards the periphery, however, many small deeply staining granules are seen, and outside these a few cells occur.

The area of degeneration, with its peripheral small-celled infiltration, passes more or less imperceptibly into an outer zone consisting of large branching cells, whose nuclei alone stain slightly ; these forming a loose meshwork of clear tissue, scattered among the interstices of which are a very varying number of small cells. These large unstaining branched cells often occur between the above mentioned processes of the central degenerated tissue. In this zone of branching cells smaller foci of degeneration are frequently seen. The boundary of the nodules is formed by a ring of fibrous tissue infiltrated with small cells. Outside this the liver cells generally appear more or less normal.

In places where a large number of deposits occur without intervening liver substance, each individual nodule exhibits the same appearances as just described in the case of the solitary ones ; the relative amount of degenerated tissue in the individual constituent deposits being apparently unchanged.

In all the deposits giant and epithelioid cells are conspicuous by their absence.

In all these deposits large numbers of tubercle bacilli are present. In the smallest, they occur regularly scattered throughout the whole tissue. In those which reveal foci of degeneration, the bacilli appear crowded in these spots. Although occurring sparingly elsewhere, they exist to, and sometimes penetrate, the encapsuling fibrous tissue, being occasionally present among the changed liver cells in the immediate proximity of the deposits. In the nodules in which degenerative processes are still more advanced, one finds bacilli scattered thickly in the peripheral parts of these areas, but are absent from the central or older regions of degeneration, as I have previously described to occur in Birds.[1] Occasionally there occur structures consisting of a central mass of hyaline degenerated tissue of varying consistency, arraged in concentric rings ; this is immediately surrounded by a broad zone of small closely disposed well-staining epithelioid cells, the whole being quite round and encapsuled by fibrous

[1] Author's "Tuberculosis in Fowls," *Trans. Path. Soc. Lond.*, 1888.

tissue. No bacilli appear to be present either in the central or peripheral regions of these structures.

The structure of the deposits in the other viscera and in the connective tissues being practically the same as the description given in the case of the liver, it is unnecessary to describe them at length.

With regard to the *Lungs*, no changes are recognized in the pulmonary tissue immediately overlying the nodules in relation to it.

The *Spleen* was full of deposits, between which but little of the intact splenic pulp remained.

In the *Pancreas* a few small deposits occur in its substance, usually in close proximity to the arterial branches.

The only well-marked deposit affecting the mucous membrane of the *Digestive Tract* appears in the lower end of the stomach, where in the substance of some of the rugæ changes of a similar nature occur, and bacilli abound here. They are also seen in some small grit-like depressions on the surface of the mucous membrane.

The small *Subcutaneous Nodules* present a central necrotic area, which is comparatively more extensive that that of the deposits in the viscera, and the small-celled infiltration around it is more abundant. The central necrosis is arranged in irregularly concentric layers, between which are roundish areas of hyaline. Scattered through this district are also clear patches of albuminous matter with small corpuscles, and occasionally blood-corpuscles and fibrine. Outside this there is considerable small-celled infiltration into the zone of clear branching cells, and here one finds hyaline patches of degeneration, and many vessels probably lymphatics containing small lymph corpuscles, and with considerable infiltration in and around their walls. Bacilli are not abundant in the layers of necrosis, but are very numerous in the roundish hyaline areas; none are seen in the clear albuminous patches occurring in the same. They are found plentifully in the hyaline foci in the external zone, being especially abundant in the tissues around the vessels.

The *Tumors* in relation to the ribs are characterized by greater vascularity, and are composed partly by large clear albuminous patches with fibrine, many small cells, and occasionally blood-corpuscles, and partly of a tissue of the same nature as described in the case of the deposits in the liver. Bacilli are again crowded in all the hyaline patches, but are absent from the albuminous ones.

Upon section through the *Aorta* and its contiguous thrombosed lymphatic, one finds considerable proliferation of the endothelium of the former along a line corresponding to the attachment of the lymphatic vessel. Bacilli abound in the muscular and serous coats of this segment of the aorta. The contents of the neighbouring vessel are of the same nature as the deposits occurring elsewhere.

From the description of this case, it is seen that we have, without a doubt, *granulomata* with central necrosis. And, moreover, in these bacilli abound which, in respect to their microscopical characters and reaction to staining reagents, would appear to be the same as those which in Man and higher animals are known as tubercle bacilli.

After this appears the diagnosis, which was given in the heading of general tuberculosis fully warranted. Whether the disease had a local origin or not, my work gives no definite information. On the other hand, so far as the seat of the tubercular deposits is concerned, there can be no doubt that those in relation to the aorta were in the lymphatic vessel; also in the viscera, they are distinctly encapsuled as if by a vessel wall; but it is noteworthy that in sections they always appear round, never oval or elongated. Again, the structure of these deposits in the viscera was the same as that of the lymphatic vessel by the aorta. So we can, on good grounds, conclude that the primary seat of the disease is found in the *lymphatic system.*

2. *Tuberculosis in Birds.*—Most of the literature on this subject deals with the disease as it occurs in the domestic fowl. The two cases I am about to describe serve to illustrate its spontaneous development in the two great classes of birds, viz., Graminivorous and Carnivorous.

The following case of a Peacock appears to be a very good example of the occurrence of tuberculosis in a graminivorous bird, being associated in this case also with bone disease, and, a point especially worthy of note, with very extensive amyloid degeneration :—

The bird was extremely emaciated, and presented several tumors of a caseous nature, often as large as walnuts, on various arts of the body. A large one was situated on the back, firmly attached to the inferior angle of the scapula. A second mass, not quite so large, was found between the planes of the muscles in this region. Another was perforating the vertical plate of the sternum towards its lower end. A mass of the same nature was seen on the inner surface of the ribs, producing their complete absorption.

The right knee-joint was beset with large masses, one the size of a walnut being attached to the anterior surface of the patella. Another, smaller, was on the inner side of the joint adherent to the capsule. In addition to these, many small nodules were found growing from the muscular tendons inserted in the neighborhood of the joint. A nodule with a loose central mass was found situated in the popliteal space, with considerable adhesion and thickening of the surrounding tissues. Upon opening the joint, little or no fluid was present in the interior. The synovial membrane was considerably metamorphosed, in places hypertrophied and pigmented, and containing numbers of small white bodies in its substance. The cartilages were soft and gelatinous. A small sinus led from the cavity of the joint into the substance of the external condyle. Upon making a section through the shaft of the femur, it was found to be exceedingly thin, only a few long spicules stretching across its interior. A large soft mass was discovered in the substance of the external condyle, with the above mentioned sinus connecting it with the joint.

There was no obvious lesion to be discovered in the other joints. A caseous mass was present in the right side of the neck, distinct and free from the œsophagus or the trachea. A deposit, the size of a pea, occurred on the external surface of the pericardium.

Another caseous mass was found projecting from the walls of the superior vena cava immediately above its entrance into the pericardium, and separated from the lumen of the vessel by a thin membrane. Upon opening the *Heart* considerable thickening of the mitral valve was seen.

The *Liver* was somewhat enlarged, its surface smooth, having a pale and waxy appearance upon section. Its substance was studded with yellowish-white miliary deposits.

The *Spleen* was also somewhat enlarged, and contained deposits which were larger than seen in the liver.

The *Kidneys* were pale, surface slightly irregular, and exhibiting a few small deposits in their substance.

The *Digestive Tract* was considerably affected with nodular enlargement of its walls; one occurred in the œsophagus, several in the duodenum, while isolated deposits were seen in the mesentery. The cæca, contrary to what is usually found in fowls, were free from deposit, they being abnormally full of fæces.

MICROSCOPICAL APPEARANCES.—In the *Liver*, in parts, the cells of the organ are considerably altered, their outlines being indistinct and their nuclei not visible. The whole tissue has a dull and opaque appearance, staining very imperfectly. Between the lobules are a few small cells, and in places vessels filled with corpuscles. All the connective tissue and wall of the vessels have an opaque and homogeneous appearance.

Scattered through the tissue are some small more or less round deposits, consisting of a central giant cell surrounded by granulation tissue. Others rather larger occur, which consist of one or more central giant cells together with large cells with indistinct nuclei; these are again surrounded by cells of an epithelioid nature with their long axes usually circularly disposed, and these by a zone of small round cells. The whole granuloma is destitute of a distinct fibrous capsule.

In most of these some degenerative change is in progress in the central area, and often is clearly beginning in the giant cells, the protoplasm many of which is undergoing hyaline degeneration in the substance of which the numerous nuclei remain for some time distinctly visible. Larger structures occur in appearance as thrombosed vessels; these contain in the centre an irregular hyaline mass, between the peripheral processes of which are numbers of giant cells. Surrounding those is a zone of granulation tissue, the granuloma being enclosed as if by a thickened vessel wall, on the inner surface of which more giant cells are sometimes seen.

It would appear that various stages from the small deposits above-mentioned to these apparently thrombosed vessels can be traced with epithelioid and sometimes giant cells, the whole appearing to be produced from a proliferating endothelium. The endothelial lining, however, of most of the vessels, as the portal vein, hepatic artery. and bile ducts, appear more or less normal.

With the iodine solution, the connective tissue and walls of the vessels, together with many of the liver cells, give the mahogany-brown color which deepens on the addition of sulphuric acid. With methyline violet the same regions stain red, the various deposits staining blue.

No bacilli could be found in any of the liver deposits.

The *Splenic* tissue is profoundly altered, the walls of the vessels being considerable thickened, and in some cases thrombosed. On examining more minutely one of these thrombi, one finds the original vessel-wall existing as a fibrous capsule, containing in places an artery with thickened walls in its substance. Within this is a broad zone of connective tissue with a few vessels and epithelioid cells, often in groups, and isolated giant cells, with here and there an irregular patch of hyaline degeneration. As we approach the centre, a zone of flattened epithelioid cells presents itself, within which again is a ring of giant cells the centre of the whole being formed by a mass of degenerated tissues in which sometimes bacilli occur.

The whole of the splenic pulp and the blood-vessels give the amyloid reactions.

In the *Kidney*, the cells of the tubules appear to be normal. Many of the glomeruli have undergone change and present an opaque appearance. A few thrombosed vessels are present. With the iodine solution all the blood-vessels—both those of the glomeruli and of the tubules—give it the amyloid reaction. With methyline violet it is seen that the amyloid change has affected especially the blood-vessels and the glomeruli, the connective tissues of the tubules, also, to a certain degree.

Upon section the mitral valve of the *Heart* presents considerable myxomatous thickening of its substance. The pericardial tumour consists of a central caseous necrosis surrounded by a broad zone of tissue much infiltrated with small cells ; around the circumference of the nercotic tissue are numbers of giant cells.

In the broad zone are numbers of groups or nests of epithelioid cells. Giant cells are also present, scattered irregularly about, the rest of the nodule being made up of small cells and fibrous tissue, in which hyaline globules are occasionally present. A few blood-vessels with changed walls are present in parts of the growth. Bacilli are present in parts of the caseous centre, but do not appear to exist in the tissue outside this. The structure of the larger tumours attached to the fibrous tissues around the bones and between the muscles is formed of a tissue of the same constitution, but with several foci of caseous degeneration of various stages, each having usually several giant cells immediately surrounding it. Bacilli do not appear to be present either in the giant cells or in the epithelioid cell-nests, and only sparingly in the clearer parts of the caseous masses.

The mass in the popliteal space is formed of the same sort of tissue, but contains many more large hyaline globules, and in its centre is a mass of small cells like that of a lymphatic tissue. No bacilli are found in this nodule.

The soft mass in the substance of the external condyle consists of a collection of granulation tissue, which in places has undergone caseous and hyaline degeneration. A few epithelioid cells are present, also a few giant cells scattered about, together with many albuminous and fatty globules. Blood-vessels filled with corpuscles are also numerous.

Sections through the synovial membrane of the diseased knee-joint show the serous membrane to be much metamorphosed. The endothelial cells appear to be replaced by a granulation tissue, the cells of which are much swollen, forming globules which give the amyloid reactions. On the surface occur many granulomata, some of which are undergoing central degeneration. These are the small white bodies which were noticed in the description of the macroscopic appearances.

From the class of canivorous birds I have taken a case occurring in a *Barn Owl*, originally from Africa, which died after a few months' captivity.

In this case, as in the former, the bird was extremely emaciated. Upon opening the abdominal cavity the surface of the viscera was seen to be studded with small white bead-like bodies, the size of large pins' heads. These were especially abundant in the neighborhood of the stomach.

The *Liver* was not much enlarged, its surface smooth, and upon section no obvious deposits were to be seen in the substance.

The *Spleen* was enlarged, surface irregular, and its substance appeared to be full of minute yellowish-white granular deposits.

In the *Pancreas* some opaque spots were seen, especially in the central regions of the organ.

The *Heart*, *Lungs*, and *Kidneys*, presented no naked eye lesions.

The mucous membrane of the *Stomach* appeared normal, but in the commencement of the duodenum a deep somewhat circular ulcer the size of 6 mm. presented itself. The edges were considerably raised and jagged, the surrounding tissues being infiltrated and thickened. Its floor showed small irregular masses of nercotic tissue, while the peritoneum over it was thickened and opaque. Lower down the intestines some small irregular patches occurred with thickened mucous membrane, but without apparent ulceration of its surface.

MICROSCOPICAL APPEARANCES.—The *Liver* vessels are numerous and filled with blood, and the whole section appears densely studded with small areas, the majority of which appear round in section ; occasionally they are seen oval or elongated. Each area is composed of a group or nest of nucleated and very granular cells of an epithelioid nature, the whole being distinctly encapsuled by bands of fibrous tissue. In some parts of the organ the deposits are so densely packed as to exhibit little or no intervening liver substance, but in no case do the individual deposits appear to fuse together, and no caseous substance is to be seen. When subjected to the methods for demonstrating the presence of tubercle bacilli, all these areas are found to be crowded, and the bacilii appear to abound in the epithelioid cells themselves. The walls of the liver vessels, viz., portal vein, hepatic artery, and bile ducts appear normal.

The whole substance of the *Spleen* is likewise densely studded with areas of the same nature as those found in the liver. All these are again crowded with bacilli, and bacilli also occur isolated and in groups, apparently in the splenic tissues outside these areas.

In the *Pancreas* the mass of secreting tissue of which appears normal, areas occur which are sometimes small and round, at other times large, but with regular outlines. These consist of a degenerated unstaining tissue containing several staining granules, and is arranged in lobuli as the secreting tissues of the organ, and in places it would appear to partly consist of changed pancreatic cells. In these areas the outlines of the original alveolæ is lost, the whole appearing much degenerated. No bacilli are present in any of these areas.

Sections through the walls of the *Stomach* reveal nothing abnormal. A section through one of the thickened patches in the small intestine exhibits a very considerable infiltration of the mucous membrane and new tissue formation. The formation consists almost entirely of groups of epithelioid cells, often round, but frequently extending in columns between the tissues of the mucous membrane, and more or less vertical to the walls of the gut. The muscular and serous coats are thickened and much changed in the region of the growth, and occasionally groups of epithelioid cells are seen scattered through the substance of the muscular and serous coats, appearing as if they found their seat in vessels. All these epithelioid cells which form the mass of the mucous membrane tumor, and exist in the muscularis and the serosa neighboring there, also likewise contain large numbers of tubercle bacilli as those cells of the splenic deposits.

A vertical section through the duodenal ulcer shows the base to be formed by the muscular coat, which is infiltrated with small cells, and granular epithelioid cells in groups. Irregular caseous masses are seen scattered through its substance, and also some occur on the surface of the ulcer. The edges of the ulcer are seen to consist of the mucous membrane much altered by infiltration of epithelioid cells. Here again bacilli abound in all the epithelioid cells, being especially numerous in the periphery of the caseous nodules, none occurring in the centres of the same. For demonstrating the presence of bacilli, I used the following methods :—

1. The sections were placed for two hours in Ziehl's solution of fuchsine and carbolic abid, and then washed with 5 per cent. sulphuric acid till all the coloring matter disappeared from the tissues, then washed in water, afterwards still further decolorised in alcohol, then through cedar oil and mounted in balsam. Instead of sulphuric acid sometime 30 per cent. nitric acid was used.

By these means I succeeded in completely decolorising the tissues, but the bacilli, even after long treatment with acid, always retained the color.

2. *Weigert's Method.*—The sections were placed for twelve hours in a solution prepared by shaking aniline oil up in distilled water and filtering, to which a few drops of methyline violet are added. From this solution the sections are removed into salt solution, and then two to three minutes in iodine solution, and then dried. After this they were completely discolorised with aniline oil, and then through xylol, and mounted in balsam. Sometimes the sections were previously stained with alum carmine.

Since the bacilli which have been stained by these methods exactly resemble in form and color those which in man and higher animals are recognized as tubercle bacilli, one has good grounds for asserting that they are identical with the latter. Accordingly, one must consider the same as tubercle bacilli, and the case as one of tuberculosis. Although I found no caseous degeneration in the deposits, even in the epithelioid cells, the nuclei and the cell boundary were well preserved, and only in the case of the duodenum did degenerative processses occur, and here an ulcer was found. Apart from the presence of genuine bacilli I found, even in the connective tissues between the deposits of epithelioid cells, a large number of round cells, so that one must reckon the whole process as lymphomatous or granulomatous tissue formation. In all cases the bacilli found in the distinctly preserved epithelioid cells, which in many respects so closely resemble the *leprous bodies* are of great interest for the whole theory of the diseases produced by bacteria.

LITERATURE.

LARCHER, *Recueil Veterinaiere*, 1871.
CRISP, *Trans. Path. Soc. Lond.* 1872 and 1875.
RIBBERT, *Deutsch. Med. Wochensch*, 1833.
JOHNE, *Deutsch Zeitsch. f. Their. Med.*, 1884.
KOCH, *Mittheilungen aus dem Kaiserlichen Gesundheit*, 1884.
SUTTON & GIBBS, *Trans. Path. Soc. Lond.*, 1884.
COVNIL & MEGNIN, *Jour. de l'Anatomie et de la Phys.*, 1885.
NOCARD, *Recueil de Medicin Veterinaie*, 1885.
DE LAMALLERÈE, *Gazette Medical*, 1886.
SIBLEY, *Trans. Path. Soc. Lond.*, 1888.

